# Supplementary material for: Autosomal Allelic Inactivation: Variable Replication and Dosage Sensitivity
Source: bioRxiv. 2025 Aug 18:2025.08.13.670061. Preprint. [Version 2] doi: 10.1101/2025.08.13.670061 (PMC12393279; doi:10.1101/2025.08.13.670061)
Supplement: Supplement 1 [file media-1.docx]

**Supplementary Figures.**

**
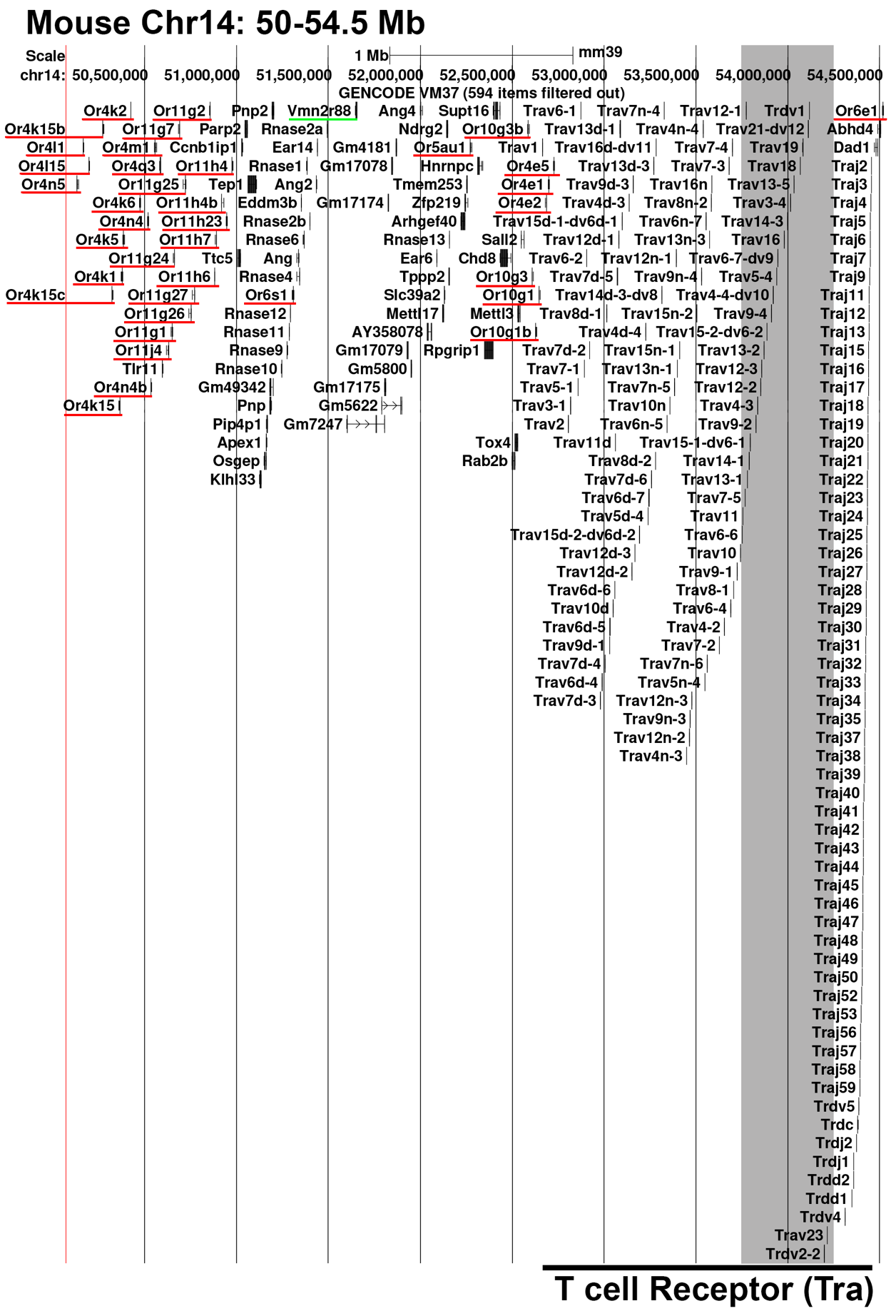
**

**Supplementary Fig. 1. UCSC Genome Browser view of the mouse VERT region (shaded) detected at the T cell receptor alpha (Tra) locus on mouse chromosome 14. The location of 37 Or genes, marked by red lines, and the vomeronasal receptor gene Vmn2r88, marked by a green line.**

**
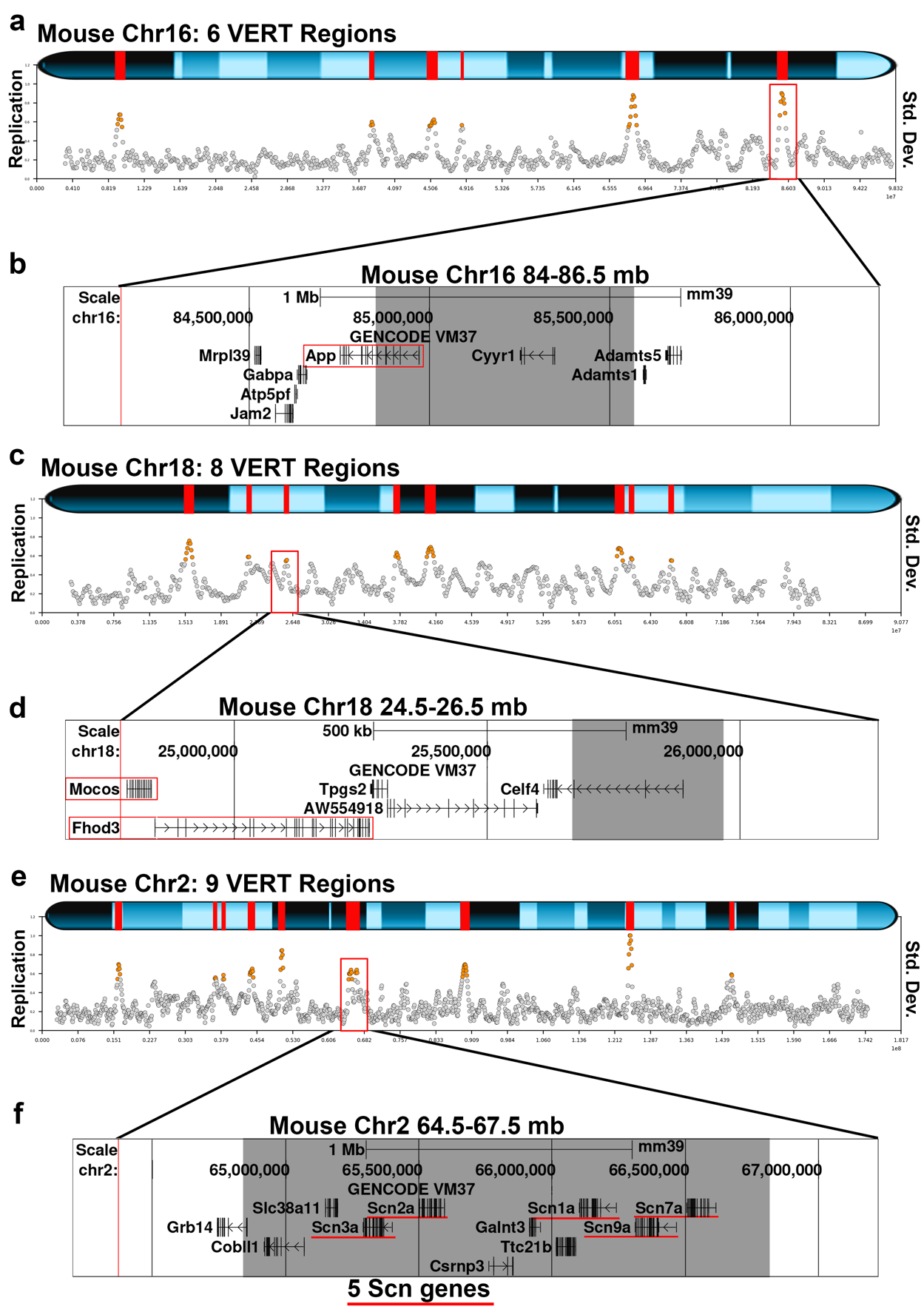
**

**Supplementary Fig. 2. Mouse syntenic regions display VERT. a, c and e) VERT regions on mouse chromosome 16, 2, and 18. The standard deviation in 50 kb windows (circles) is shown. Outlier windows from pre-B cell clones are highlighted in orange. The VERT regions highlighted by red boxes are expanded in panels b, d, and f. b) UCSC Genome Browser view of the VERT region in a above, highlighting the location of the mouse App gene (red box). The shaded area represents the VERT region. d) UCSC Genome Browser view of the VERT region in panel c, highlighting the location of the mouse Mocos and Fhod3 genes (red boxes). The shaded area represents the VERT region. f) UCSC Genome Browser view of the VERT region in panel c, highlighting the location of five mouse Scn genes (red boxes). The shaded area represents the VERT region.**
